# Surveillance of Fresh Artisanal Cheeses Revealed High Levels of *Listeria monocytogenes* Contamination in the Department of Quindío, Colombia

**DOI:** 10.3390/pathogens10101341

**Published:** 2021-10-17

**Authors:** Elizabeth Jaramillo-Bedoya, Yenny Alexandra Trujillo-Alzate, Iván Darío Ocampo-Ibáñez

**Affiliations:** 1 Laboratorio de Salud Pública Departamental, Secretaría de Salud Departamental del Quindío, Gobernación del Quindío, Armenia 630008, Colombia; lizzyjaramillo88@gmail.com (E.J.-B.); yennyalexandratrujilloalzate2020@hotmail.com (Y.A.T.-A.); 2 Research Group of Microbiology, Industry and Environment (GIMIA), Faculty of Basic Sciences, Universidad Santiago de Cali, Cali 760035, Colombia

**Keywords:** foodborne diseases, *Listeria monocytogenes*, artisanal fresh cheeses

## Abstract

Listeriosis is a foodborne disease caused by *Listeria monocytogenes*. Because outbreaks of listeriosis are associated with the ingestion of contaminated dairy products, surveillance of artisanal cheeses to detect the presence of this microorganism is necessary. We collected three types of artisanal non-acid fresh cheese (Campesino, Costeño, and Cuajada) from 12 municipalities of the Department of Quindío, Colombia. *L. monocytogenes* was identified using VIDAS^®^ and confirmed with API^®^ Listeria Rapid Kit. *L. monocytogenes* was detected in 104 (53.6%) of the 194 artisanal fresh-cheese samples analyzed. The highest percentages of contamination were detected in Salento (90.9%), Calracá (65.5%), Armenia (64.9%), and Filandia (50%). A significant association between municipality and contamination with *L. monocytogenes* was identified. However, no association could be established between the type of cheese and the occurrence of the bacterium. This is the first study on the presence of *L. monocytogenes* in artisanal fresh cheeses sold in the municipalities of the Department of Quindío, and the findings revealed very high percentages of contaminated samples. The presence of *L. monocytogenes* in artisanal cheeses remains a public health threat in developing countries, especially Colombia, where existing legislation does not require the surveillance of *L. monocytogenes* in food.

## 1. Introduction

*Listeria monocytogenes* is an intracellular Gram-positive pathogenic bacterium that causes listeriosis, a serious foodborne disease in humans that can occur in a non-invasive or an invasive form [1]. Non-invasive listeriosis (febrile listerial gastroenteritis) is a mild form of the disease and has symptoms such as fever, diarrhea, headache, and myalgia [1,2]. Invasive listeriosis is more severe and may affect specific groups of the population such as pregnant women, infants, the elderly, and people with co-morbidities. Its symptoms include fever, myalgia, septicemia, meningoencephalitis, and miscarriages [1,2]. Pregnant women are a high-risk group as they can vertically transmit the bacteria to the fetus through the placenta, and perinatal listeriosis can lead to miscarriage, fetal death, septicemia at birth, or neonatal meningitis [3]. The incidence of listeriosis is low, with 0.1–10 cases per million people per year depending on the country and region [1]. However, the mortality rate associated with the infection is high, which makes it a major public health concern worldwide; for example, mortality rates of 20%–30% have been reported for invasive listeriosis [1]. Over the past decades, many outbreaks of listeriosis associated with the ingestion of contaminated food have been reported globally in different regions and countries, including the United States and several nations in South America, Europe, and Africa [2,4,5,6]. Among them was a large-scale and deadly outbreak that occurred in South Africa between 2017 and 2018 owing to the ingestion of processed meat contaminated with *L. monocytogenes* [4,7]. This was the largest listeriosis outbreak to date, with more than 1000 cases and approximately 200 deaths [4,7]. In Colombia, data on prevalence, incidence, and outbreaks of listeriosis are scarce because it is not a reportable disease [8,9]. Nevertheless, in recent years, some listeriosis outbreaks involving a few cases have been documented in different regions of Colombia, namely 19 confirmed cases of invasive listeriosis in Cali in 1999, a 9-year-old boy with manifestation of listeriosis-related meningitis in 2009, two cases confirmed by laboratory tests in Nariño in 2017, four cases in Bogotá in 2018, and three cases of *L. monocytogenes* infection in Antioquia in 2019 [8,9,10].

Consuming food contaminated with high levels of *L. monocytogenes* is the main route for acquiring listeriosis [1,2,11,12,13,14]. Most sporadic cases and outbreaks of listeriosis worldwide have been directly associated with the intake of contaminated fresh or processed food [4,11,15,16,17,18,19,20]. *L. monocytogenes* is able to adapt to a wide variety of niches and conditions; therefore, it can contaminate diverse food products [21]. This bacterium can multiply under diverse conditions commonly used for the preservation of fresh or processed food, both at room temperature and under refrigeration, at extreme pH or even at high salinity levels [21,22]. Particularly, raw or processed products of animal origin, such as milk, meat, dairy products, or sausages, are frequently contaminated with *L. monocytogenes* [16,18,23,24]. This contamination could be attributed to the fact that animals are often asymptomatic carriers of *L. monocytogenes*, which can lead to cross-contamination during the manufacturing of derived products [25]. For example, *L. monocytogenes* can be a part of the udder microbiota of dairy animals, sometimes causing clinical or subclinical mastitis. This microbial presence can lead to the contamination of milk by the excretion of bacteria over long periods of time during milking and, consequently, to contamination of milk products [26].

In view of the above findings, *L. monocytogenes* can pose a serious threat to the dairy industry [27]. Many listeriosis outbreaks worldwide have been associated with the ingestion of contaminated cheese, raw (unpasteurized) milk, or ice cream [1,2]. Specifically, soft cheeses, such as fresh cheese, white cheese, and Camembert cheese produced with unpasteurized milk are high-risk products and, therefore, constitute an important route for the spread of listeriosis [1,2,28]. It has been estimated that the probability of *L. monocytogenes* infection is 50–160 times higher when the said cheeses are produced with unpasteurized milk compared to the production with pasteurized milk [2]. Although pasteurization kills bacteria, fresh cheeses made with pasteurized milk are still susceptible to contamination with *L. monocytogenes* if the conditions of the production facilities are unsanitary [2,4]. The problem is exacerbated in artisanal fresh cheese production settings, where surfaces are not properly sanitized and artisanal cheese makers have bare-hand contact with the raw material and final products [29,30]. Specifically, in Colombia, there is a wide variety of non-acid fresh cheeses, such as Campesino, Costeño. and Cuajada, which are produced in artisanal settings under the stated conditions [31,32]. The production of all these typical Colombian non-acid fresh cheeses involves a milk coagulation process. Then, for each type of cheese, there are differences in the steps subsequent to whey draining, such as the addition of high amounts of salt for preservation (Costeño cheese) or grinding and kneading (Campesino cheese) [31,32]. Governments and industry around the world perform surveillance constantly to ensure the microbiological safety of cheeses [2,4,27,33,34], including artisanal fresh cheeses [35,36]. In Colombia, the current legislation does not establish mandatory surveillance for *L. monocytogenes* in fresh or processed food [8,9,37]. Nevertheless, the presence of *L. monocytogenes* has been reported in artisanal fresh cheeses produced and sold in the departments of Antioquia, Córdoba, Norte de Santander, and Valle del Cauca [9,37,38,39,40,41]. Presently, the prevalence of this bacterium in cheeses manufactured and marketed in the Department of Quindío in Colombia remains unknown. Therefore, in view of the lack of mandatory surveillance in the current Colombian legislation, this cross-sectional study aimed to determine the presence of *L. monocytogenes* in three typical Colombian artisanal fresh cheeses sold in cheese retail stores in the 12 municipalities of the Department of Quindío in 2021. This is the first study on the presence of *L. monocytogenes* in artisanal fresh cheeses sold in the municipalities of this department of Colombia.

## 2. Results

**Figure 1:**
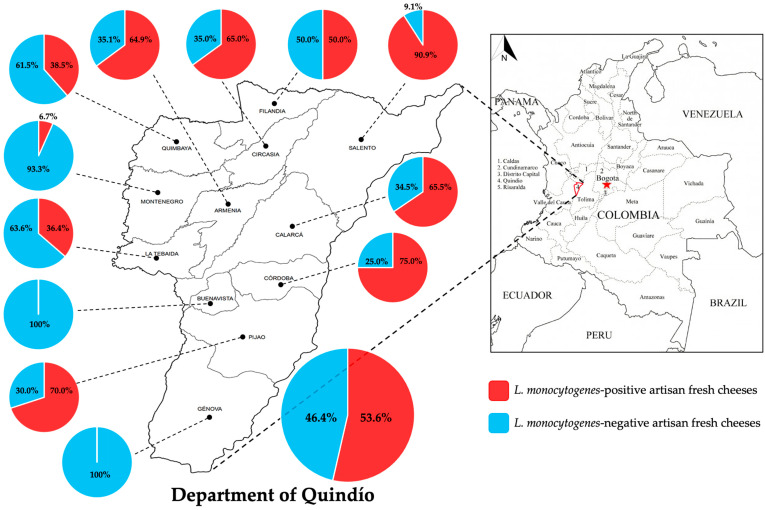
Prevalence of *Listeria monocytogenes* in different municipalities of the Department of Quindío.

This cross-sectional study aimed to determine the presence of *L. monocytogenes* in three typical Colombian artisanal fresh cheeses sold in the municipalities of the Department of Quindío during 2021. A total of 194 samples of non-acid artisanal fresh cheeses were collected from 128 cheese retail stores in the 12 municipalities of the Department of Quindío. *L. monocytogenes* was detected in 104 samples (53.6% prevalence) from 10 of the 12 municipalities studied (Figure 1). The prevalence of *L. monocytogenes* varied significantly among the municipalities, from 6.7% in Montenegro to 90.9% in Salento (Figure 1). Furthermore, Salento, Córdoba, and Pijao presented high prevalence values of 75% and 70%, respectively (Figure 1). In addition, high prevalence of *L. monocytogenes* was observed in artisanal fresh cheeses from the municipalities with the highest population densities in the department, namely Armenia (64.9%), Calarcá (65.5%), Circasia (65%), Quimbaya (38.5%), and La Tebaida (36.4%) (Figure 1). The lowest prevalence values were found in Buenavista (0%), Génova (0%), and Montenegro (6.7%) (Figure 1). Of the 104 samples positive for *L. monocytogenes*, the highest numbers of contaminated samples were found in Armenia (24) and Calarcá (19) (Table 1). Among the samples negative for *L. monocytogenes*, Filandia and Montenegro accounted for the highest proportions of non-contaminated samples of 16.7% and 15.6%, respectively (Table 1). Comparison analysis showed significant differences in the behavior of positive and negative samples for *L. monocytogenes* in relation to the municipality (Table 1). 

The three typical Colombian non-acid fresh cheeses analyzed in this study, Campesino, Costeño, and Cuajada, presented similar prevalence values for *L. monocytogenes* (Table 2). Costeño cheese was the most frequently contaminated product (57.1%). Among the positive samples, Cuajada cheese showed the highest percentage of contamination (52.9%), followed by Campesino cheese (39.4%) (Table 2). Fisher’s test showed no significant differences in the behavior of the samples positive and negative for *L. monocytogenes* in relation to the type of cheese analyzed (Table 2).

## 3. Discussion

This work is the first cross-sectional study on the prevalence of *L. monocytogenes* in artisanal fresh cheeses traded in all the municipalities of the Department of Quindío, Colombia. The prevalence values observed in this study were extremely high, with a maximum contamination level of 53.6%, which is much higher than the average prevalence reported for cheeses in different countries in Africa, Asia, North America, South America, and Europe [42,43,44]. In this regard, the overall prevalence of *L. monocytogenes* in cheeses produced in Europe is 2.3%. Our values are comparable to those reported for cheeses from Portugal (46%) and Italy (55%) [42,44,45], and significantly higher than those reported for other countries in South America (Brazil, 11%) [44,46] and North America (the United States, 2%; Mexico, 35.7%) [44,47,48]. Moreover, the overall prevalence observed in our study was higher than that previously reported in other departments of Colombia, such as Córdoba (0%) [41], Cundinamarca (13.3%) [39], Norte de Santander (16.3%) [40], Valle del Cauca (27%) [38], and Antioquia (33.1%) [37]. Since the present Colombian legislation does not require mandatory surveillance of *L. monocytogenes* in cheese [37], there is no legal limit for the presence of the bacterium in this type of food. Nonetheless, the overall prevalence of *L. monocytogenes* in cheeses from Quindío found in our study is remarkably high, particularly the prevalence in some municipalities such as Salento (>90%) (Figure 1). In contrast, no contaminated cheeses were detected in other municipalities such as Buenavista and Génova (prevalence of 0%). Significant differences were observed in the prevalence of *L. monocytogenes* relative to the municipality, which indicates that there is a relationship between the presence of the bacterium in non-acid artisanal fresh cheese and the municipality of origin (Table 1).

Artisanal fresh cheeses are among the food products of greatest public health concern because of their high risk of contamination by *L. monocytogenes* [1,2,28,29,30]. The main reasons for this risk include the conditions of the production settings and the microbiological quality of the milk used for their manufacture [29,30]. In particular, soft cheeses made from unpasteurized milk are an important vehicle for the spread of *L. monocytogenes* [1,2,28,49]. Three typical Colombian cheeses were analyzed in this study, namely Campesino, Costeño, and Cuajada. The contamination level in these cheeses ranged from 53.2% to 57.1%. No significant differences were found in the behavior of positive and negative samples for *L. monocytogenes* in relation to the type of cheese analyzed, i.e., there was no relationship between the type of cheese and the presence of the bacterium. This observation could be attributed to the manufacturing process of artisanal non-acid fresh cheeses, which is similar for Campesino, Costeño, and Cuajada cheeses. All these three types of fresh-curd cheeses do not undergo ripening; consequently, there are not particular conditions which allow the survival of the bacteria in a differential way. The contamination levels found in the present study were higher than the overall average prevalence of *L. monocytogenes* previously reported in soft cheeses (2.4%–4.4%) [42,43,44,50]. Nevertheless, our results are comparable with previous reports on the prevalence of *L. monocytogenes* in soft and semi-soft cheeses, such as the cheeses sold in Greece (40%) [33,44], Castelo Branco cheese from Portugal (46%) [44,45], and blue-veined cheese from Italy (55%) [42,43,44,50,51]. The contamination levels described here were higher than those reported for fresh cheeses [42,43,44]. In this regard, the average prevalence of *L. monocytogenes* reported for European fresh cheeses was 0.8% [42]. The occurrence of contamination in fresh Latin-style cheeses in North America and South America ranged from 0.0% to 37.5%, whereas in African and Asian countries, the contamination levels were as high as 4.2% and 9.2%, respectively [44,52]. In Europe, the presence of *L. monocytogenes* has been reported with different prevalence values in different types of fresh cheeses, such as cream cheese in Italy (1.9%) [53], cheeses manufactured and sold by local producers in Italy (with contamination levels ranging from 3.5% to 12.9%) [54,55,56], and cheeses sold in retail stores in Spain (1.3%) [57] and Austria (4%) [58]. In contrast, no contamination with *L. monocytogenes* [44] was found in cheeses such as Burrata (Italy) [59], Mozzarella (Italy) [53], Ricotta (Italy) [55], homemade cheeses (Austria) [58], and cheeses produced and sold in dairy farms (Sweden) [60]. The prevalence values of this bacterium in fresh cheeses produced in North and South American countries were much lower than those reported here [44]. In this respect, Mexican fresh cheeses such as panela, adobera, and cottage cheese showed contamination levels of 37.5%, 18.8%, and 6.7%, respectively [44,47,61]; fresh cheeses traded in the United States near the Mexican border presented a prevalence of 2% for *L. monocytogenes* [48], and Minas frescal cheese from Brazil showed contamination levels of up to 11% [46]. Our study reports the highest prevalence values of *L. monocytogenes* in artisanal fresh cheeses in Colombia. The contamination level observed for Campesino cheese (53.2%) was much higher than that previously reported for the departments of Valle de Cauca (36.8%) [38] and Norte de Santander (6%) [40]. Likewise, the percentage of Costeño cheese samples positive for *L. monocytogenes* in the Department of Quindío found in this study (57.1%) was much higher than the one noted in Cali (Department of Valle del Cauca) (8.3%) [38] and in the Department of Córdoba (0%) [41]. In addition, the observed occurrence of *L. monocytogenes* in Cuajada cheese was higher than the one previously reported in Cali (36.6%) [38] and Norte de Santander (3.6%) [40]. 

Our results revealed high levels of *L. monocytogenes* contamination in the artisanal non-acid fresh cheese sold in the Department of Quindío. Despite the lack of information on the manufacturing conditions of the fresh cheeses analyzed, it is known that many of the products traded in the Department of Quindío are produced in rural areas in an artisanal manner. Despite that *L. monocytogenes* is relatively resistant to heat, this bacteria is not able to survive the pasteurization process at high temperatures even in case of short-term exposure [22,62,63]. Therefore, the contamination observed in this study could be attributed to the use of raw (unpasteurized) milk, improperly pasteurized milk, or a mixture of contaminated raw milk and pasteurized milk [1,2,28,64]. Moreover, the presence of *L. monocytogenes* in the analyzed cheese samples could be due to contamination occurring after pasteurization of the milk or during cheese production. Artisanal cheese-making facilities may not have appropriate sanitary conditions, the surfaces may not be properly sanitized, and the artisanal cheese makers may have bare-hand contact with the raw material and final products [2,4,29,30]. This contact could lead to cross-contamination during the manufacturing of cheese or even at later handling stages [65]. The prevalence of *L. monocytogenes* among the food handlers is quite high in Colombia. A study conducted in 10 Colombian departments (Antioquia, Atlántico, Boyacá, Caquetá, Córdoba, Cundinamarca, Meta, Nariño, Santander, and Valle del Cauca) reported a 10.4% prevalence of *L. monocytogenes* among the meat and dairy food handlers [66]. Hence, it is important to emphasize that the manufacturing process and handling stages of the artisanal non-acid fresh cheeses analyzed in this study were not considered.

This study included estimates of the prevalence of *L. monocytogenes* in artisanal non-acid fresh cheeses traded in the Department of Quindío in a relatively small sample of cheeses, which constitutes a limitation. However, it is important to highlight that this is the first study conducted for this purpose and that markedly high prevalence values of *L. monocytogenes* were found in this region. Considering the fact that the current legislation in Colombia does not require the mandatory surveillance of *L. monocytogenes* in food, and in view of the serious consequences that infection by this bacterium can have on human health, this study intends to raise awareness of the need to implement surveillance and control mechanisms for *L. monocytogenes* in the production and trading of artisanal fresh cheeses.

## 4. Materials and Methods

### 4.1. Sampling Site and Sampling 

This study was conducted in May and June 2021 in the Department of Quindío, located in Central–West Colombia between 04°04′41″ N–04°43′18″ N and 75°23′41″ W–75°53′56″ W. Samples were collected randomly from different cheese retail stores in the 12 municipalities of the departments of Quindío, namely, Armenia, Buenavista, Calarcá, Circasia, Córdoba, Filandia, Génova, La Tebaida, Montenegro, Pijao, Quimbaya, and Salento. A comparative cross-sectional design with a non-probability convenience sampling of 194 cheese samples (150–200 g each) was used in this study. Three classes of non-acid fresh cheeses (Campesino, Costeño, and Cuajada) sold in the Department of Quindío were analyzed. No information was available on the place of origin and manufacturing conditions of these cheeses. The collection was done as part of the laboratory-based surveillance of the Departmental Public Health Laboratory of Quindío. 

### 4.2. Isolation and Identification of Listeria monocytogenes 

The presence of *L. monocytogenes* in the cheese samples was determined in accordance with the ISO standards and Food and Drug Administration guidelines [67,68]. Detection was performed with the VIDAS^®^ *Listeria monocytogenes* II commercial kit (bioMérieux, Marcy l’Etoile, France) according to the manufacturer’s instructions. Briefly, 25 g of artisanal fresh cheese was homogenized, selectively pre-enriched for *Listeria* spp. in 225 mL Half-Fraser broth (Biomerieux) and incubated at 30 °C for 24 h. Then, 1 mL of this suspension was transferred to 10 mL Fraser broth (Biomerieux) and incubated at 30 °C for 24 h. From this final suspension, 500 μL were used for the detection in the VIDAS^®^ system. Those suspensions containing *L. monocytogenes* were cultured in Oxford and PALCAM (bioMérieux) selective media at 35 °C for 24 h. and the obtained colonies were confirmed through biochemical tests using the API^®^ Listeria kit (bioMérieux) according to the manufacturer’s instructions. All assays were performed in triplicate, and at least two independent assays were performed for each cheese sample.

### 4.3. Statistical Analysis

The frequency distribution of the qualitative variables was determined by univariate and bivariate analyses using contingency tables. The distribution of *L. monocytogenes* relative to the municipality of origin of the artisanal fresh cheese was determined. Statistical significance was evaluated using Fisher’s exact test with a 0.05 level of significance. All analyses were performed using the statistical package R-Project software Version 1.1.463.

## 5. Conclusions

Our estimations of the prevalence of *L. monocytogenes* confirm that the artisanal non-acid fresh cheeses produced and traded in the municipalities of the Department of Quindío are frequently contaminated by this microorganism. These results suggest that the conditions for the production and trading of these cheeses in the department do not comply with the optimal standards for safety and sanitation. Furthermore, in view of the fact that many of the cheeses marketed nationwide are manufactured in artisanal settings, this type of study should be scaled up and replicated in all departments of Colombia. The results observed in the present study highlight the urgent need to implement surveillance and control mechanisms for *L. monocytogenes* in the production and trading of food at the departmental and national levels. The risk that this pathogen poses to human health, especially for high-risk groups such as pregnant women, infants, the elderly, and immunocompromised individuals, should be borne in mind while devising surveillance and control strategies.

## Figures and Tables

**Table 1:** Proportion of positive and negative samples for *Listeria monocytogenes* at the different sampling sites.

Municipality ^1^	Positive Samples		Negative Samples		Total Samples
	N	%	N	%	
Armenia	24	23.1	13	14.4	37
Buenavista	0	0.0	2	2.2	2
Calarcá	19	18.3	10	11.1	29
Circasia	13	12.5	7	7.8	20
Córdoba	6	5.8	2	2.2	8
Filandia	15	14.4	15	16.7	30
Génova	0	0.0	8	8.9	8
La Tebaida	4	3.8	7	7.8	11
Montenegro	1	1.0	14	15.6	15
Pijao	7	6.7	3	3.3	10
Quimbaya	5	4.8	8	8.9	13
Salento	10	9.6	1	1.1	11
Total	104	100	90	100	194

^1^ Significance level for the behavior of positive and negative samples for *L. monocytogenes* in relation to the municipality (*p*-Value = 0.0001 < 0.05). Abbreviations: N, Number of samples.

**Table 2:** Proportion of positive and negative samples for *Listeria monocytogenes* in different types of artisanal non-acid fresh cheese.

Type of Cheese ^1^	Positive Samples		Negative Samples		Total Samples	Prevalence
	N	%	N	%		(%)
Queso Campesino	41	39.4	36	40.0	77	53.2
Queso Costeño	8	7.7	6	6.7	14	57.1
Queso Cuajada	55	52.9	48	53.3	103	53.4
Total	104	100	90	100	194	

^1^ Significance level for the behavior of positive and negative samples for *L. monocytogenes* in relation to the type of cheese (*p*-Value = 1.0000 > 0.05). Abbreviations: N, Number of samples.

## Data Availability

The datasets generated during and/or analysed during the current study are available from the corresponding author on reasonable request.
